# Effects of isometric vs. dynamic resistance training on muscle performance and body composition: Protocol for a pilot study

**DOI:** 10.1016/j.jsampl.2025.100108

**Published:** 2025-06-20

**Authors:** Morteza Ghayomzadeh, Alex Natera, Angelo Sabag, Brock Cooper, Glen M. Davis, Daniel A. Hackett

**Affiliations:** a Discipline of Exercise and Sports Science, Sydney School of Health Sciences, Faculty of Medicine and Health, The University of Sydney, Camperdown, NSW, Australia; b Sport Science, New South Wales Institute of Sport, Sydney Olympic Park, NSW, Australia

**Keywords:** Isometric training, muscle hypertrophy, Muscular strength, Resistance training

## Abstract

**Background:**

Isometric resistance training (ISO-RT) has gained renewed attention for its potential to elicit muscular adaptations and enhance athletic performance. Unlike dynamic resistance training (DYN-RT), ISO-RT involves no joint movement or eccentric loading, making it particularly suitable for individuals with joint pathologies or those undergoing rehabilitation. Despite increasing interest, the comparative effectiveness of ISO-RT versus DYN-RT across various outcomes, including strength, hypertrophy, endurance, and recovery, remains inadequately explored.

**Aims:**

This study aims to evaluate and compare the effects of multi-angle ISO-RT and traditional DYN-RT on muscle performance, body composition, and recovery-related indicators in healthy adults.

**Methods:**

In this pilot randomised controlled trial, 20 healthy adults (≥18 years) will be randomly assigned to either the ISO-RT or DYN-RT group (n ​= ​10 per group). Both groups will complete a full-body resistance training program twice weekly for six weeks. The key distinction lies in the execution of the chest press and leg press exercises—performed isometrically in the ISO-RT group and dynamically in the DYN-RT group. All outcomes will be assessed at baseline and post-intervention.

**Analysis:**

Primary outcomes include dynamic and isometric strength. Secondary outcomes encompass muscular power, dynamic and isometric endurance, body composition (via dual-energy X-ray absorptiometry), muscle oxygenation (via near-infrared spectroscopy), and subjective recovery indicators such as sleep quality and delayed onset muscle soreness. Between-group comparisons will be conducted using appropriate inferential statistical tests to determine effect estimates and feasibility metrics.

**Discussion/implications:**

This trial will offer preliminary insights into the physiological and perceptual adaptations elicited by ISO-RT versus DYN-RT. The findings will inform the design of larger-scale trials and contribute to developing tailored, evidence-based resistance training guidelines for both clinical and athletic populations.

**Key points:**

1. Isometric resistance training (ISO-RT) has been shown to enhance muscular adaptations. However, it is unclear whether ISO-RT leads to greater, comparable, or lesser muscular adaptations than traditional dynamic resistance training (DYN-RT).
2. This study protocol aims to advance understanding of the comparative efficacy of multi-angle ISO-RT vs. DYN-RT in enhancing muscular strength, hypertrophy, and both isometric and dynamic muscular endurance. It will also assess muscle oxygenation and subjective recovery markers such as sleep quality and muscle soreness. The findings will provide preliminary insights into the physiological and perceptual responses to these training modalities, informing future research and the design of larger-scale trials.

## 1 Introduction

Resistance training (RT) is widely recognised as an effective modality for improving muscle strength and hypertrophy. Resistance training can be broadly categorised into dynamic and isometric muscle actions. Dynamic muscle actions involve joint movement and can be further divided into concentric (shortening) and eccentric (lengthening) phases [1]. In contrast, isometric muscle actions involve muscle contraction without visible joint movement. Isometric RT (ISO-RT) has a long-standing history, with anecdotal reports suggesting its use by strength and conditioning practitioners to enhance athletic performance through trial and error [2].

Isometric RT first gained scientific recognition in 1953 when Hettinger and Müller demonstrated that daily 6-s applications of ISO-RT could increase isometric strength by approximately 5 ​% per week [3]. In this study, participants performed submaximal isometric contractions (∼67 ​% maximal voluntary contraction - MVC) once daily for 6 ​s, with loads adjusted weekly to maintain relative intensity. This landmark study spurred interest in ISO-RT, leading subsequent researchers to explore its potential applications for health and performance [2,4]. Compared to dynamic RT (DYN-RT), ISO-RT offers unique adaptations. Isometric training can enhance strength at specific points in the range of motion (ROM), making it useful for targeting the weak spots in the ROM (i.e., sticking points), that may limit athletic performance [2]. Additionally, ISO-RT is often the preferred training method during rehabilitation for individuals with joint pathologies due to its ability to minimise mechanical stress on the joints. Its lower energy demand and the absence of eccentric muscle contractions also allow for faster recovery, enabling more frequent training and potentially greater cumulative adaptations over time [5].

Although promising, ISO-RT has been criticised for its joint-angle specificity, with strength gains often confined to the trained positions and showing limited transfer to adjacent angles [2]. Additionally, its capacity to induce muscle hypertrophy has been questioned - likely due to the absence of eccentric contractions and limited mechanical stimulus across a full ROM [2,4]. However, the perception that ISO-RT leads to inferior hypertrophic adaptations compared to DYN-RT may largely reflect methodological shortcomings in earlier studies, particularly the use of insufficient training volume and intensity. Given that both volume and intensity are key drivers of hypertrophy, it is plausible that well-designed ISO-RT protocols (featuring adequate load and volume) can elicit muscular growth comparable to DYN-RT. Nevertheless, few studies have directly compared hypertrophic outcomes between ISO-RT and DYN-RT under matched training conditions. Much of the existing ISO-RT research relies on suboptimal protocols and within-subject or unilateral limb designs, which are susceptible to confounding factors such as cross-education effects and baseline strength asymmetries between limbs [[6], [7], [8], [9], [10]]. Interestingly, emerging evidence suggests that multi-angle ISO-RT may promote more robust and generalised adaptations than traditional single-angle approaches, potentially mitigating these limitations and enhancing external validity [7].

Beyond muscle strength and hypertrophy, ISO-RT and DYN-RT may elicit distinct physiological and perceptual responses that influence training feasibility, recovery, and long-term adherence. For example, differences in local metabolic stress and fatigue accumulation could affect post-exercise recovery [11], yet these factors remain underexplored. Muscle oxygenation, which reflects real-time oxygen delivery and utilisation, is one such understudied variable. Although near-infrared spectroscopy (NIRS) offers a non-invasive means of assessing this, direct comparisons between ISO-RT and DYN-RT using NIRS are currently limited. Equally important are markers of recovery, such as muscle soreness and sleep quality, which can affect training tolerance and compliance over time. Despite their relevance, the influence of muscle soreness and sleep quality on adaptations and adherence across RT modalities remains poorly defined. Together, these gaps underscore the need for research that moves beyond traditional outcome measures to capture a more comprehensive understanding of responses to ISO-RT and DYN-RT.

To address these gaps, the current pilot study investigates the feasibility and preliminary efficacy of a novel multi-angle ISO-RT protocol in comparison to DYN-RT. A comprehensive set of outcomes will be evaluated, including muscular strength, hypertrophy, isometric and dynamic muscle endurance, fatigue recovery, muscle oxygenation, muscle soreness, and sleep quality. The findings will offer foundational insights to guide the design of future large-scale trials and support evidence-based integration of ISO-RT into both athletic and clinical RT settings.

## 2 Aims

This pilot study explores the impact of a 6-week multi-angle ISO-RT program on physiological and perceptual outcomes, including muscular strength, hypertrophy, dynamic and isometric muscular endurance, muscle oxygenation, fatigue recovery, sleep quality, and muscle soreness.

## 3 Methods

### 3.1 Study design

**Fig. 1:**
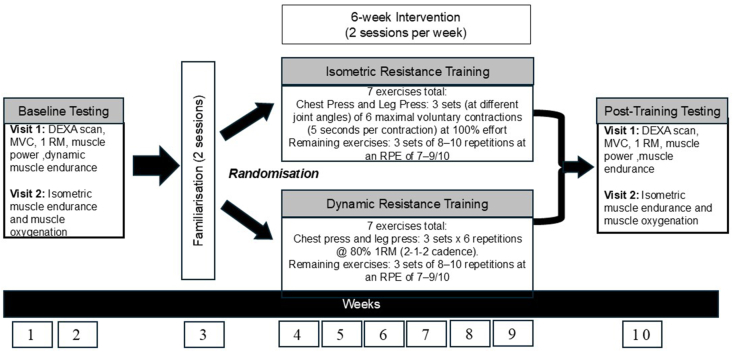
Intervention timeline.

This intervention will employ a randomised controlled trial (RCT) design. Eligible participants will be randomly assigned to the groups of ISO-RT (n ​= ​10) and DYN-RT (n ​= ​10). They will perform 6 weeks of training under the supervision of researchers. The protocol consists of a 2-week baseline assessment and a 1-week familiarisation phase comprising two sessions. Following the familiarisation, the participants of both groups will undergo their respective 6-week RT interventions, during which participants will attend two sessions per week. Following the intervention period, the study will conclude with a one-week follow-up assessment. This schedule results in a total study duration of 10 weeks, during which each participant will complete 17 visits (see Fig. 1). Ethical approval for this study was granted by the University of Sydney Human Research Ethics Committee (HREC 2024/HE001016). Informed consent will be obtained from all participants before they participate in the study.

### 3.2 Participant allocation and intervention protocol

Eligible participants will undergo a 1-week familiarisation period before being randomly assigned to one of two 6-week full-body RT programs. Both groups will consist of two training sessions per week, following identical protocols for most exercises. Both groups will train twice weekly using an identical protocol for five out of the seven prescribed exercises: seated row, biceps curl, triceps pushdown, knee extension, and knee flexion. The training programs for both groups will be identical, except for the chest press and leg press exercises. In the ISO-RT group, the chest press and leg press will be performed isometrically. Participants will be instructed to exert maximal effort during each repetition to reflect real-world applications of ISO-RT better. Each repetition will consist of a 5-s contraction at 100 ​% effort executed at three distinct joint angles (lower third, mid-range, and upper third of the ROM). Joint angles for the chest and leg press will be determined using a goniometer during the initial familiarisation session and replicated consistently throughout the intervention and during baseline and follow-up testing sessions. In contrast, the DYN-RT group will perform the chest press and leg press exercises dynamically using a 2-1-2 repetition cadence (2-s concentric, 1-s pause, 2-s eccentric) at 80 ​% of their one-repetition maximum (1RM). In both groups, these exercises will be performed for three sets of six repetitions, with a 2-min rest between sets. The remaining five exercises will be identical across both groups, performed through the full ROM using a controlled 5-s repetition tempo. The target intensity will correspond to a perceived exertion of 7–9 out of 10 on the Borg CR10 scale, with inter-set rest intervals of 90–120 ​s. Prior to the chest press and leg press, participants in both groups will complete two standardised warm-up sets consisting of five repetitions at ∼50 ​% and ∼70 ​% of their maximal strength levels (1RM for the DYN-RT group and MVC for the ISO-RT group).

Table 1 provides details of the training intervention protocols.

**Table 1:** Training Intervention Protocols (performed twice per week).

Exercise Order	ISO-RT Group	DYN-RT Group
**1 & 2**	Isometric CP and LP, 3 sets^a^ x 6 repetitions (5 ​s per repetition) @ effort to produce 100 ​% MVC. 120-sec rest b/w sets	Dynamic CP and LP. 3 sets x 6 repetitions (2-1-2 cadence) at 80 ​% 1RM. 120-sec rest b/w sets
**3**–**7**^b^	Exercises: SR, TP, KE, KF, BC. 3 sets x 8–10 repetitions @ RPE of 7–9/10. 90–120 ​s rest b/w sets	

Abbreviations: b/w ​= ​between; CP = Chest Press; KE = Knee Extension; KF = Knee Flexion; LP ​= ​Leg Press; sec ​= ​seconds; SR = Seated Row; TP ​= ​Triceps Pushdown; BC= Biceps Curl, 1RM ​= ​one-repetition maximum; RPE ​= ​rate of perceived exertion.

a With each set, it will perform at a different angle (lower, middle, upper range of motion - ROM).

b Participants will follow an identical exercise training protocol in both groups, except for the chest press and leg press, which will be performed isometrically in the ISO-RT group and dynamically in the DYN-RT group.

### 3.3 Training equipment and isometric force monitoring setup

**Fig. 2:**
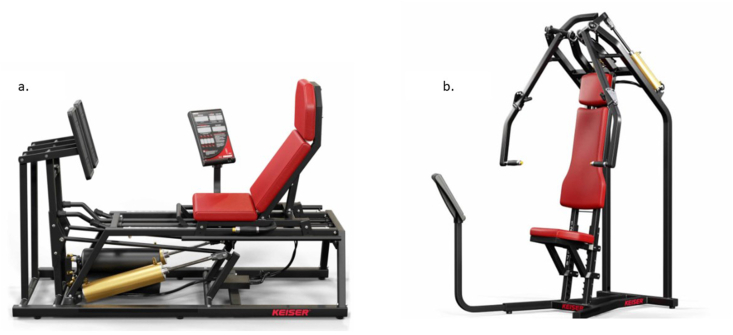
Keiser A400 pneumatic resistance machines: (a) Biaxial leg press and (b) Biaxial seated chest press.

**Fig. 3:**
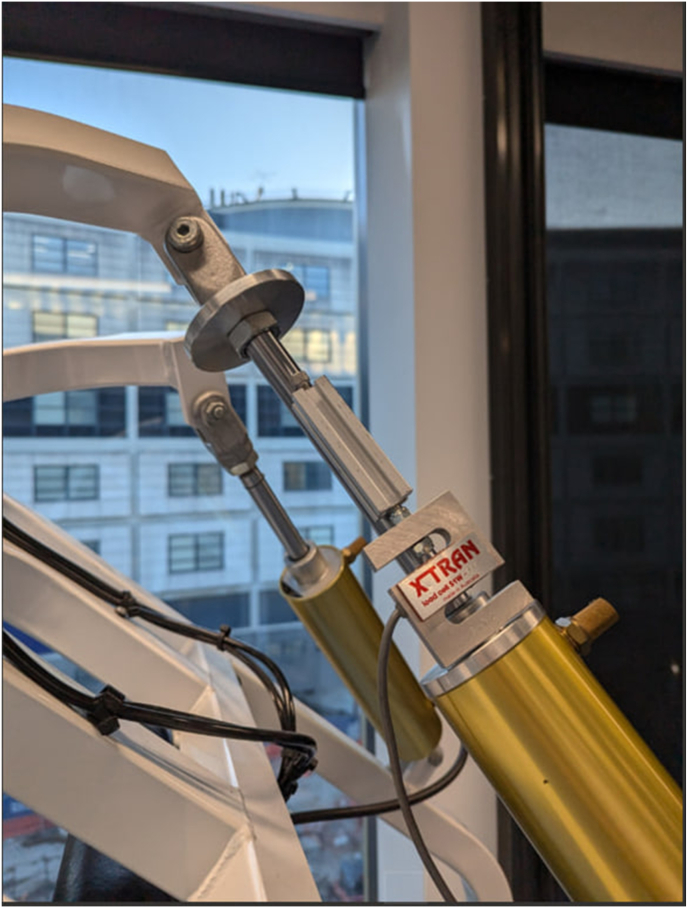
Load cell integrated with the custom-engineered actuator system on the Keiser pneumatic resistance machine.

All resistance exercises except bicep curls, which will be performed with free weights, will be executed using Keiser A400 pneumatic resistance machines (Keiser Sports Health Equipment, Fresno, California, USA), which enable precise control of resistance and movement velocity through pneumatic load modulation (see Fig. 2). These machines are commonly used in both clinical and high-performance settings due to their smooth resistance profiles and real-time feedback capabilities. For the isometric chest press and leg press exercises, a custom-engineered actuator system has been developed to fix the machine arms at specific joint angles, effectively transforming the dynamic Keiser machines into isometric testing and training platforms. The actuator assembly, which mounts directly to the pneumatic cylinder housing, securely restricts joint movement at predefined angles using a high tolerance locking mechanism (see Fig. 3). This setup allows participants to perform isometric contractions at consistent joint positions across all sessions.

**Fig. 4:**
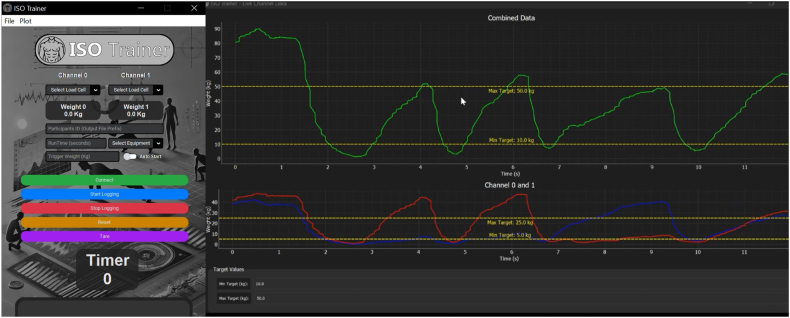
ISOTrainer V1.1 interface displaying real-time isometric force output with visual feedback and target thresholds from dual-channel load cell inputs.

To quantify force output during isometric contractions, each actuator is fitted with an AMA XTRAN® strain gauge-based load cell (Australia). The transducer transmits force data via USB-C to a laptop running ISOTrainer V1.1, a custom-built software application developed specifically for this study (see Fig. 4). The ISOTrainer interface allows real-time monitoring and recording of applied force through two independent channels (Channel 0 and Channel 1). A key feature of ISOTrainer is its live visual feedback module, which displays real-time force traces on a dual–axis graph interface (see Fig. 4). Participants will receive immediate visual feedback on their contraction force and for the muscle endurance test will receive feedback relative to individualised target thresholds (e.g., 80 ​% MVC), represented by upper and lower yellow limit lines. This supports consistent effort regulation and engagement during repeated isometric trials and training sets. All force data are time-stamped and automatically saved in structured output files for subsequent analysis. This integrated hardware-software setup allows for high-fidelity, reproducible measurement of isometric force production, while enhancing participant engagement and test standardisation through visual biofeedback.

### 3.4 Eligibility criteria

Participants will be eligible for this study if they meet the following conditions: they are males or females aged 18 years or older; have not engaged in structured RT within the past three months; do not have any clinical conditions; have no musculoskeletal injuries; are able to provide written informed consent and complete study measures in English; and are willing and able to attend both pre- and post-intervention exercise testing and twice weekly training sessions across an 6-week period at the University of Sydney's Susan Wakil Health Building (Sydney, NSW, Australia).

### 3.5 Participant recruitment

Participants for this study will be recruited from the broader Sydney region of Australia. Recruitment efforts will include general announcements on the university research study website, postings on noticeboards, sign-up sheets, and engagement by research team members who have no prior relationship with potential participants to minimise any risk of coercion. Within the University community, recruitment will be further facilitated through e-bulletins such as “Yamma” and “Volunteer for Research Studies.” Additionally, the study will be advertised on social media platforms, including Facebook and Instagram, with all promotional materials prominently featuring the research team's contact information. Interested individuals will be able to reach out directly to the research team for more details. Participation in the study will be entirely voluntary, and all outreach materials will explicitly highlight this to ensure informed and voluntary engagement.

### 3.6 Pre-testing familiarization session

To minimise learning effects that could otherwise inflate post-intervention performance outcomes, all participants will complete a dedicated familiarisation session prior to baseline testing. This session is particularly important given that participants are inexperienced with structured RT and the pneumatic resistance equipment used in this study. The session will include guided, hands-on instruction covering all outcome measures (i.e., 1RM dynamic strength, MVC, muscle power, and muscular endurance). Participants will be instructed on proper lifting techniques, breathing control, equipment handling, and movement cadence specific to each test (e.g., 2-1-2 tempo for dynamic lifts, 5-s holds for isometric efforts). They will also be introduced to equipment feedback systems, including real-time force and power displays, to ensure familiarity with test procedures. The goal of this session is to ensure that participants are confident, consistent, and technically proficient during all baseline assessments. This approach enhances the accuracy, reliability, and reproducibility of performance data across timepoints.

### 3.7 Data collection

An identification code will be used to identify participants' data. The study's principal investigator will create the identification code, and these details will not be shared with the rest of the research team. This approach ensures the confidentiality and anonymity of participants' data, protecting their privacy in accordance with ethical research standards. Only the study's chief investigator will have access to the code to link participant identities if necessary for follow-up or data verification. The research staff will enter the data gathered into REDCap Digital, a secure web application hosted by the University of Sydney that builds and manages databases [12].

## 4 Outcome measures

### 4.1 Dynamic strength (1RM)

Dynamic muscular strength will be assessed using a 1RM test for both the chest press and leg press (Keiser Sports Health Equipment, Inc., Fresno, CA, USA). In accordance with National Strength and Conditioning Association (NSCA) and American College of Sports Medicine (ACSM) protocols, participants will first complete a general warm-up followed by a specific warm-up consisting of multiple submaximal repetitions at a light-to-moderate intensity (corresponding to a rating of perceived exertion [RPE] of 4–6 on the Borg CR10 scale) to reduce the risk of injury and ensure neuromuscular readiness. Following the warm-up, participants will perform single repetitions with progressively increased loads. A successful lift will be defined as the ability to complete the full ROM with proper technique and control. Rest intervals of 2–3 ​min will be provided between attempts to allow adequate recovery, as recommended by NSCA guidelines. If a participant reports an RPE ≥9 (on the 0–10 scale), the rest period may be extended up to 4 ​min. The test will conclude when the participant is unable to complete the lift for two consecutive attempts at a given load. The highest load successfully lifted will be recorded as the 1RM. All 1RM testing will be conducted under strict safety protocols, including proper machine setup and clear verbal instructions. Standardised verbal encouragement will be provided throughout the testing to promote maximal effort.

### 4.2 Isometric strength

Maximal isometric strength will be evaluated for both the chest press and leg press using MVCs at the same joint angles applied during the ISO-RT intervention: one-third (just before lockout), two-thirds (mid-range), and the end of the eccentric phase. Testing will be conducted using a custom-built isometric rig equipped with a calibrated force transducer, with real-time force output displayed on a monitor (as described in section 3.3). At each angle, participants will perform three maximal 5-s contractions, with a 2-min rest interval between attempts to ensure adequate recovery and minimise fatigue. If a participant reports an RPE of 9 or higher on the Borg CR10 scale, the rest period may be extended to 3 ​min. The highest recorded force at each angle will be used as the participant's MVC. Joint angles will be measured using a goniometer during the familiarisation session and replicated consistently across all testing sessions. Standardised verbal encouragement will be provided during each contraction to promote maximal effort.

### 4.3 Muscle power

Muscle power will be assessed using Keiser pneumatic resistance equipment with K400 electronics. Participants will perform a single explosive contraction at incremental loads ranging from 20 ​% to 80 ​% of their most recent 1RM (in 10 ​% increments) for the chest press and leg press. A 60-s rest will be provided between efforts, with one practice trial at 20 ​% 1RM before progressing to higher loads. Peak power outputs will be recorded at each load.

### 4.4 Dynamic muscular endurance

Dynamic muscular endurance for the chest press and leg press will be evaluated using Keiser pneumatic resistance equipment, set at a standardised load of 60 ​% 1RM. Participants will perform as many repetitions as possible with proper form, adhering to the 2-1-2 ​s cadence (2-s concentric, 1-s pause, 2-s eccentric) used during training sessions. The test will be terminated when the participant is unable to complete a repetition with proper form or maintain the prescribed cadence for two consecutive repetitions. Recorded metrics will include the total number of repetitions, total work performed, as well as the velocity and power generated during the first and last repetitions.

### 4.5 Isometric muscular endurance

Isometric muscular endurance for the chest and leg press will be assessed using Keiser pneumatic RT equipment. Participants will perform intermittent isometric contractions set at 80 ​% of their predetermined MVC. Equipment adjustments will ensure participants’ joints are positioned at the midpoint of the concentric phase. The protocol comprises repeated cycles of a 5-s isometric contraction at 80 ​% MVC followed by a 5-s rest interval. The ISOTrainer live visual feedback module will enable participants to maintain accurate and consistent contraction intensity throughout the test. The test will terminate when participant-generated force falls below 50 ​% MVC during two consecutive repetitions. Recorded metrics will include the total number of successful contractions (repetitions) and the total time to exhaustion (in seconds).

### 4.6 Body composition

Body composition (i.e., whole-body and appendicular muscle mass) will be measured in whole-body scan mode at baseline and post-intervention using Dual-energy X-ray Absorptiometry (DEXA) (Lunar Prodigy, GE Medical Systems, Madison, WI) [13]. All scans will be conducted under standard conditions by a licensed co-investigator. Participants will be instructed to adhere to pre-scan guidelines, including a minimum 12-h fast from solid food, a 6-h fast from liquids, and refraining from exercise for at least 24 ​h. The in-built enCORE analysis software (version 13.60.033; GE Healthcare) will be used to calculate total lean mass, fat mass, and regional composition (e.g., appendicular lean mass for upper and lower limbs). To minimise variability and enhance the accuracy of measurements, participants will be instructed to follow standardised pre-scan procedures, including fasting from solid food for at least 12 ​h, refraining from liquid intake for 6 ​h, avoiding vigorous physical activity for at least 24 ​h, and voiding their bladder and bowels within 60 ​min prior to the scan. Adherence to these protocols will be confirmed verbally by study staff prior to each scanning session.

### 4.7 Sleep quality

Sleep quality will be assessed using the Single-Item Sleep Quality Scale (SQS), a validated tool designed to capture overall subjective sleep experience over the past 7 days [14]. Participants will rate their sleep quality on an 11-point numerical scale ranging from 0 (“terrible”) to 10 (“excellent”), considering various aspects such as sleep duration, ease of falling asleep, night-time awakenings, early morning awakenings, and perceived restfulness upon waking. This measure provides a simple yet reliable index of perceived sleep quality, which may influence recovery and training responsiveness [14].

### 4.8 Muscle soreness

Perceived muscle soreness will be measured using a 100-mm Visual Analogue Scale (VAS), a widely accepted method for quantifying delayed onset muscle soreness (DOMS). Participants will be asked to indicate the intensity of soreness they feel in the chest and leg muscles, respectively, by marking a point on the horizontal line where 0 ​mm represents “no pain” and 100 ​mm represents “severe, intolerable pain.” The VAS will be administered at 24- and 48-h post-training following each exercise session throughout the intervention period. This approach allows for the tracking of exercise-induced muscle soreness and its potential modulation across different RT modalities [15].

## 5 Statistical analysis

### 5.1 Sample size

As this is a pilot study with an exploratory focus, no formal a priori power calculation has been conducted, as the primary objective is not to test hypotheses but to estimate effect sizes, assess outcome variability, and evaluate the feasibility of the intervention protocol. Moreover, due to the limited availability of previous studies employing comparable designs (i.e., directly comparing isometric and dynamic RT on strength and body composition), there is insufficient data to support a reliable power analysis at this stage. Based on methodological recommendations for pilot trials, a total sample size of 20 participants (10 per group) is considered appropriate for this phase of investigation. This is consistent with established guidance suggesting that 10–12 participants per group are sufficient to estimate key design parameters such as effect size variability, adherence, and procedural feasibility in small-scale studies [16,17]. Although the sample is not powered to detect statistically significant between-group differences, post hoc sensitivity analysis indicates that, with an alpha of 0.05 and 80 ​% power, the study would be able to detect very large effect sizes (Cohen's d ​≥ ​1.2). While detecting effects of this magnitude would suggest strong intervention potential, the primary aim of this study is to generate preliminary data to inform the design and powering of an adequately powered trial in the future.

### 5.2 Data analysis plan

The effects of ISO-RT vs. DYN-RT on primary outcomes (isometric and dynamic muscular strength and power) and secondary outcomes (muscle hypertrophy, muscular endurance, and muscle oxygenation) will be analysed using a two-way repeated measures ANOVA, with time (pre- and post-intervention) as the within-subject factor and group (ISO-RT vs. DYN-RT) as the between-subject factor. Where significant main effects or interactions are observed, Bonferroni-adjusted post hoc tests will be used to determine pairwise differences. To compare between-group percentage changes in outcome variables (e.g., strength, power, isometric and dynamic muscular endurance, hypertrophy) a one-way ANOVA will be conducted, followed by Bonferroni correction for multiple comparisons. All analyses will be conducted using SPSS software (Version 29.0; IBM Corp., Armonk, NY, USA), with statistical significance set at p ​< ​0.05.

## 6 Discussion

This study outlines a rigorously designed pilot trial aimed at comparing the effects of multi-angle ISO-RT and DYN-RT on muscle performance, body composition, and recovery-related responses. While DYN-RT has traditionally been the gold standard for improving muscular strength and hypertrophy, multi-angle ISO-RT offers a promising alternative by potentially overcoming the joint-angle specificity of conventional isometric protocols. Nonetheless, high-quality controlled trials are needed to determine whether these theoretical advantages translate into superior or comparable adaptations in practice. This trial is among the first to directly compare these two training modalities using a comprehensive battery of outcome measures. In addition to primary outcomes, the study evaluates secondary physiological markers such as muscle hypertrophy, power, endurance, and muscle oxygenation. In the current study, muscle oxygenation of the vastus lateralis will be assessed before and after dynamic and isometric muscular endurance tests using mNIRS technique, which is a non-invasive, real-time technique for monitoring local tissue oxygenation and blood volume dynamics. NIRS enables continuous quantification of changes in oxygenated and deoxygenated hemoglobin, offering valuable insights into the muscle's metabolic and vascular responses to exercise. By evaluating these parameters during both endurance protocols and standardising the signal through post-test maximal isometric contractions, this method allows for direct comparison of the physiological demands imposed by ISO-RT and DYN-RT. These data may help clarify how different resistance training modalities influence local muscle oxygenation and recovery capacity.

The inclusion of subjective recovery metrics, including perceived muscle soreness and sleep quality, provides a more holistic view of training responsiveness, offering insights into how these modalities may differentially impact recovery, tolerability, and long-term adherence. Together, these data will yield preliminary effect estimates and feasibility indicators to inform the design and implementation of larger-scale, adequately powered studies. In doing so, the findings will contribute to the growing evidence base needed to develop individualised and context-specific RT guidelines.

### 6.1 Strengths and potential impact

This study outlines a rigorously designed pilot trial aimed at comparing the effects of multi-angle ISO-RT and DYN-RT on muscle performance, body composition, and recovery-related responses. While DYN-RT has traditionally been the gold standard for improving muscular strength and hypertrophy, multi-angle ISO-RT offers a promising alternative by potentially overcoming the joint-angle specificity of conventional isometric protocols. Nonetheless, high-quality controlled trials are needed to determine whether these theoretical advantages translate into superior or comparable adaptations in practice.

This trial is among the first to directly compare the muscular adaptations of ISO_RT vs DYN_RT by using two independent groups. The study employs a comprehensive battery of outcome measures, with primary endpoints including dynamic and isometric strength. Secondary outcomes encompass key physiological markers such as muscle hypertrophy, power, muscular endurance, and muscle oxygenation. Collectively, these data will yield preliminary effect estimates and feasibility indicators, informing the design and implementation of future large-scale, adequately powered trials. Ultimately, the findings will contribute to the evidence base needed to guide the development of individualised and context-specific ISO-RT programs.

## Credit author statement

**Morteza Ghayomzadeh:** Conceptualisation; Methodology; Writing - original draft; Writing - review and editing. **Angelo Sabag:** Conceptualisation; Writing - review & editing. **Brock Cooper:** Design and engineered the required setup for the intervention; Writing - review & editing. **Glen Davis:** Conceptualisation; Writing - review & editing**; Alex Natera:** Conceptualisation; Methodology; **Daniel Hackett:** Conceptualisation; Writing - review & editing; Writing - original draft.

## Declaration of competing interest

The authors declare no conflicts of interest related to this study protocol. The development of the protocol was conducted independently and was not influenced by any financial or personal relationships. The authors affirm that the study design is original, adheres to ethical standards, and was developed to ensure methodological rigor and reproducibility.

## References

[1] McArdle W.D., Katch F.I., Katch V.L. (2006).

[2] Lum D., Barbosa T.M. (2019). Brief review: effects of isometric strength training on strength and dynamic performance. Int J Sports Med.

[3] Hettinger T., Müller E.A. (1953). Muskelleistung und muskeltraining. Arbeitsphysiologie.

[4] Oranchuk D.J., Storey A.G., Nelson A.R., Cronin J.B. (2019). Isometric training and long-term adaptations: effects of muscle length, intensity, and intent: a systematic review. Scand J Med Sci Sports.

[5] Carvalho L., Junior R.M., Barreira J., Schoenfeld B.J., Orazem J., Barroso R. (2022). Muscle hypertrophy and strength gains after resistance training with different volume-matched loads: a systematic review and meta-analysis. Appl Physiol Nutr Metabol.

[6] Jones D.A., Rutherford O.M. (1987). Human muscle strength training: the effects of three different regimens and the nature of the resultant changes. The Journal of physiology.

[7] Folland J.P., Hawker K., Leach B., Little T., Jones D.A. (2005). Strength training: isometric training at a range of joint angles versus dynamic training. J Sports Sci.

[8] Davies J., Parker D.F., Rutherford O.M., Jones D.A. (1988). Changes in strength and cross sectional area of the elbow flexors as a result of isometric strength training. Eur J Appl Physiol Occup Physiol.

[9] Rich C., Cafarelli E. (2000). Submaximal motor unit firing rates after 8 wk of isometric resistance training. Med Sci Sports Exerc.

[10] Weir J.P., Housh T.J., Weir L.L., Johnson G.O. (1995). Effects of unilateral isometric strength training on joint angle specificity and cross-training. Eur J Appl Physiol Occup Physiol.

[11] Curovic I. (2025). The role of resistance exercise-induced local metabolic stress in mediating systemic health and functional adaptations: could condensed training volume unlock greater benefits beyond time efficiency?. Front Physiol.

[12] Willers C., Lynch T., Chand V., Islam M., Lassere M., March L. (2022). A versatile, secure, and sustainable all-in-one biobank-registry data solution: the A3BC REDCap model. Biopreserv Biobanking.

[13] Shepherd J.A., Ng B.K., Sommer M.J., Heymsfield S.B. (2017). Body composition by DXA. Bone.

[14] Snyder E., Cai B., DeMuro C., Morrison M.F., Ball W. (2018). A new single-item sleep quality scale: results of psychometric evaluation in patients with chronic primary insomnia and depression. J Clin Sleep Med.

[15] Price D.D., McGrath P.A., Rafii A., Buckingham B. (1983). The validation of visual analogue scales as ratio scale measures for chronic and experimental pain. Pain.

[16] Lancaster G.A., Dodd S., Williamson P.R. (2004). Design and analysis of pilot studies: recommendations for good practice. J Eval Clin Pract.

[17] Hertzog M.A. (2008). Considerations in determining sample size for pilot studies. Res Nurs Health.

